# Identification and characterization of a factor Va-binding site on human prothrombin fragment 2

**DOI:** 10.1038/s41598-019-38857-4

**Published:** 2019-02-21

**Authors:** Alexander P. Friedmann, Anatoli Koutychenko, Chengliang Wu, James C. Fredenburgh, Jeffrey I. Weitz, Peter L. Gross, Ping Xu, Feng Ni, Paul Y. Kim

**Affiliations:** 1 Thrombosis and Atherosclerosis Research Institute, Hamilton, Ontario Canada; 2 Department of Medical Sciences, McMaster University, Hamilton, Ontario Canada; 3 Department of Medicine, McMaster University, Hamilton, Ontario Canada; 4 Department of Biochemistry, McGill University, Montreal, Quebec Canada; 5 Advanced Biological Analytics Section, Department of Downstream Processing and Analytics, Human Health Therapeutics Research Centre, National Research Council Canada, Montreal, Quebec Canada

## Abstract

The fragment 2 domain (F2) of prothrombin and its interaction with factor (F) Va is known to contribute significantly to prothrombinase-catalyzed activation of prothrombin. The extent to which the F2-FVa interaction affects the overall thrombin generation, however, is uncertain. To study this interaction, nuclear magnetic resonance spectroscopy of recombinant F2 was used to identify seven residues within F2 that are significantly responsive to FVa binding. The functional role of this region in interacting with FVa during prothrombin activation was verified by the FVa-dependent inhibition of thrombin generation using peptides that mimic the same region of F2. Because six of the seven residues were within a 9-residue span, these were mutated to generate a prothrombin derivative (PT6). These mutations led to a decreased affinity for FVa as determined by surface plasmon resonance. When thrombin generation by an array of FXa containing prothrombinase components was monitored, a 54% decrease in thrombin generation was observed with PT6 compared with the wild-type, only when FVa was present. The functional significance of the specific low-affinity binding between F2 and FVa is discussed within the context of a dynamic model of molecular interactions between prothrombin and FVa engaging multiple contact sites.

## Introduction

The immediate need to minimize blood loss upon vascular injury is met with the formation of a stable fibrin clot that is triggered by the rapid activation of the blood coagulation cascade. A common theme in such an emergency response is the involvement of multi-component complexes composed of an enzyme, a protein cofactor, the lipid surface and a divalent cation such as the prothrombinase and both the extrinsic and intrinsic tenase complexes^1^. The prothrombinase complex, formed in the penultimate step of the coagulation cascade, is composed of the serine protease factor (F) Xa and the protein cofactor FVa, assembled on negatively charged phospholipid membranes (*e*.*g*. activated platelets)^2,3^ and is mediated by calcium ions^1^. This complex accelerates prothrombin activation by several orders of magnitude^4^, leading to a rapid accumulation of thrombin and conversion of circulating fibrinogen into a fibrin clot.

It has been established that the full assembly of coagulation complexes can increase their catalytic efficiencies by several orders of magnitude, as in the cases of the prothrombinase^5^ and the full tenase complexes^6^. Underlying this, enzyme/protein components of each macromolecular complex appear to have many points of contact with one another with varying binding affinities^7,8^. For example, FVa has discrete binding contacts with each of the four structural domains of prothrombin^9–12^, and in turn prothrombin interacts with the multi-domain enzyme FXa^13,14^. This example is in concert with the concept of multivalent molecular assembly^15^ which has begun to emerge as a model for intracellular machineries^16^. One essential feature of multivalent assembly is the requirement for transient and weak component interactions to both confer highly dynamic molecular interactions^15^, and respond to “on-demand” regulation^16–18^. This emerging picture of macromolecular machinery is reminiscent of the dynamics of coagulation enzyme assembly and subsequent thrombin generation, as shown in a number of studies from our laboratory^19,20^ and others^21^. This dynamic regulation may also be required for physiological functions of thrombin generation other than haemostasis^22–24^.

**Figure 1:**
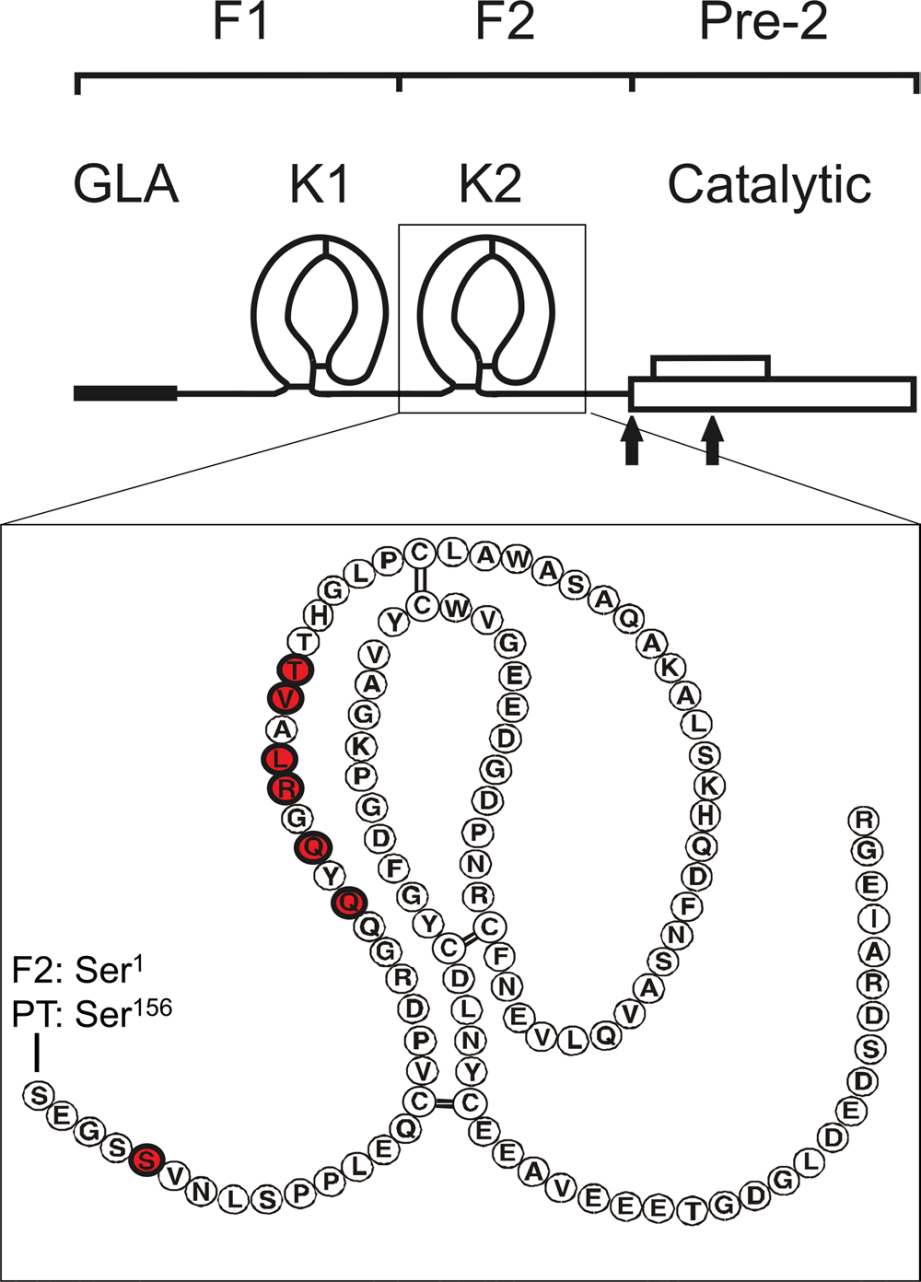
The structural organization of prothrombin. Prothrombin consists of fragment 1 (F1), which contains the γ-carboxyglutamic (GLA) and kringle 1 (K1) domains, fragment 2 (F2), which contains the kringle 2 (K2) domain, and the catalytic domain, which is also referred to as prethrombin-2 (Pre-2). The cleavage sites for activation of prothrombin by FXa (Arg271 and Arg320) are indicated with arrows. The close-up image of the K2 domain is shown with its amino acid sequence. Ser1 of K2 corresponds to Ser156 of full-length prothrombin sequence numbering. The seven residues identified using heteronuclear NMR in our study are highlighted in red.

While high-affinity interactions have been studied and characterized extensively, other, weaker but equally important interactions have largely been ignored for coagulation complexes due to the challenges in studying their binding properties. We sought to investigate weaker interactions in the context of the prothrombinase complex, specifically the binding interactions between FVa and the F2 domain of prothrombin (Fig. 1). These low-affinity protein-protein interactions in prothrombinase^9,14,25^ have been suggested to play an important functional role in the turnover of prothrombin^26^. We further characterize the binary interactions between FVa and prothrombin, in particular that between the heavy chain of FVa (FVa-HC) and the F2 domain of prothrombin. Multi-dimensional nuclear magnetic resonance (NMR) was used to identify the residues within the F2 domain that are perturbed upon FVa-HC binding, which were then subsequently mutated. The functional consequences of the loss of these F2 residues were then investigated. Significance of the specific binding between F2 and FVa-HC is discussed in the context of a dynamic network of domain interactions between prothrombin and FVa as part of the catalytic machinery of prothrombinase.

## Results

### Identifying F2 residues involved in binding FVa-HC

**Figure 2:**
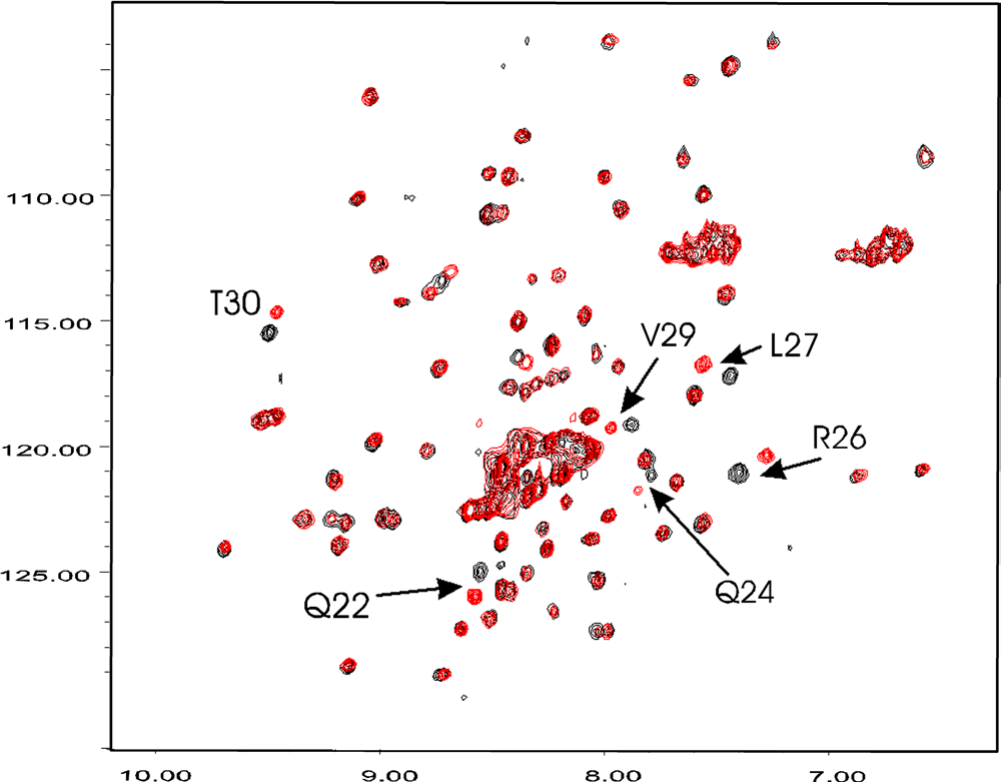
Overlay of the ^15^N HSQC spectra (at 500 MHz) of free F2 and FVa heavy chain-titrated F2. The HSQC spectra were recorded in 90% H_2_O and 10% D_2_O with 50 mM sodium phosphate at pH 7.0 and at 35 °C. The protein concentrations were 0.3 mM for F2 and 0.03 mM for the FVa heavy chain (FVa-HC). HSQC peaks of F2 most shifted by FVa are labelled by the assigned F2 residues. The arrows point to the center of resonance displacement between the free F2 (*black*) and F2 complexed with FVa (*red*). Peak displacements of the affected F2 residues decreased gradually (not shown) as the FVa-HC concentration varied between 0.03 mM and 0.01 mM.

Recombinant F2 domain of prothrombin and purified F2 from human plasma prothrombin (Fig. 1) exhibited essentially the same functional activities for enhancing the activation of prethrombin-2 by prothrombinase. Proton and ^15^N/^1^H HSQC NMR spectra of recombinant F2 exhibited progressive changes with increasing concentrations of FVa-HC from 10 to 30 μM (Fig. 2), indicating a binding affinity (K_d_) in the low to middle μM range. ^15^N/^1^H HSQC spectra at the highest (30 μM) FVa-HC concentration collected for the ^15^N-labeled F2 showed that a majority of the ^15^N/^1^H cross-peaks for F2 were not affected by the presence of the FVa-HC (Fig. 2). However, there were 7 HSQC peaks with significant chemical shift perturbations for the backbone and/or side-chain ^15^N and/or ^1^H resonances of F2, with six of the seven located in the N-terminal Gln22-Thr30 segment of the kringle. For example, Gln24 and Leu27 (residues 179 and 182 in prothrombin numbering) displayed changes of ~0.10 and ~0.08 ppm for the proton chemical shift (Δδ_H_) and ~0.9 and ~0.7 ppm for the amide nitrogen resonance (Δδ_N_), respectively. Residues Arg26 and Thr30 (prothrombin residues 181 and 185) also exhibited perturbed resonances with Δδ_H_~0.08 and Δδ_H_~0.03; Δδ_N_~0.8 and Δδ_N_~0.9 ppm, respectively, while residues Ser5 and Val29 (prothrombin residues 160 and 184) were somewhat perturbed upon titration of the FVa-HC. Importantly, F2 residues Gln22-Thr30 (prothrombin residues 177–185) perturbed by FVa all reside on a highly exposed surface loop of the kringle structure of F2 in the non-covalent complexes of F2 with Phe-Pro-Arg-chloromethylketone-inhibited thrombin (FPRck-thrombin)^27,28^, prethrombin-2^29^, as well as prethrombin-1 (prothrombin des F1) (Fig. 1)^30^. Therefore, all the highly perturbed resonances likely correspond to residues located at or near the binding interface^31^ within the binary molecular complex of F2 with FVa-HC.

### Functional interactions of F2-derived peptides

To test whether the clustered residues on F2 of prothrombin identified by NMR represent the FVa-HC binding site, a 20-residue peptide that encompasses this region (F2P-(16-36) (prothrombin residues 171–191) was synthesized, with Cys36 replaced with a serine^32^. Compared with full-length F2, this truncated peptide did not exhibit pronounced proton resonance perturbations with up to 6-fold molar excess of FVa-HC. However, it did show a significant enhancement of nuclear Overhauser effects (NOEs) when comparing their two-dimensional NOE spectra (NOESY) in the absence and presence of the FVa-HC. Specifically, residues Val29 and Thr30 (Val184 and Thr185 in prothrombin numbering) in F2P-(16-36) exhibited the strongest FVa-HC-induced NOEs, *i*.*e*. transferred NOEs^33^ within the region of intact F2 that is the most responsive to FVa-HC binding in H-^15^N HSQC experiments (Fig. 2). Taken together, these data suggest that the 20-mer region contains, at least in part, the residues identifiable by NMR as a potential FVa-HC binding site.

**Figure 3:**
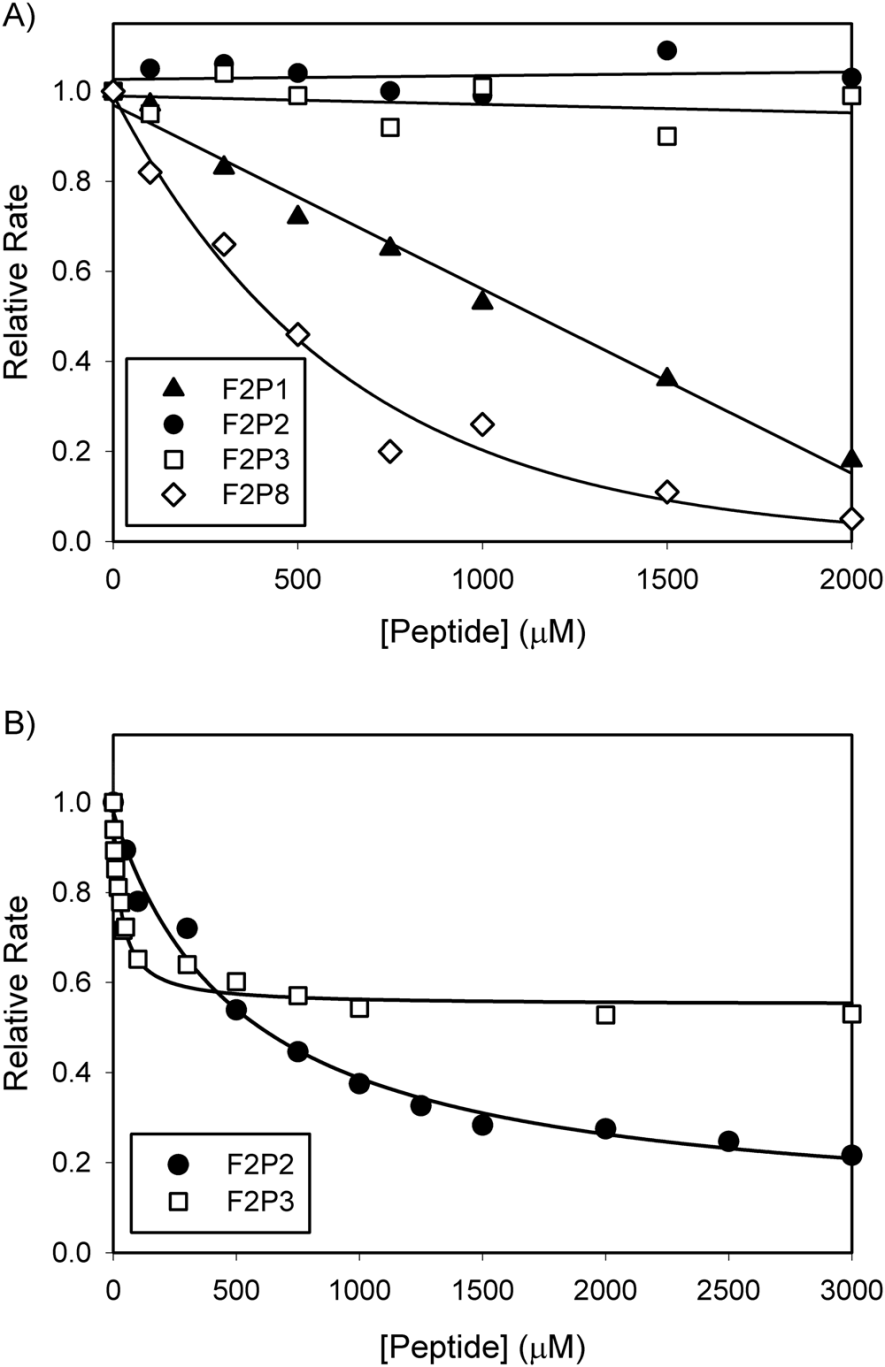
Inhibition of FXa-mediated prothrombin activation by synthetic peptides derived from F2. (**A**) Relative rates of prothrombin activation in the presence of 300 nM prothrombin, 5 mM CaCl_2_, 5 μM DAPA, 50 μM PCPS, and F2P1 (*triangles*), F2P2 (*circles*), F2P3 (*squares*) or F2P8 (*diamonds*) at varying concentration are plotted with respect to inhibitor concentrations. Reactions were started by addition of 20 nM FXa. (**B**) Prothrombin activation was carried out as above, but in the presence of 5 nM FVa and F2P2 (*circles*), or F2P3 (*squares*) at varying concentrations. Reactions were initiated by addition of 0.1 nM FXa. Non-linear regression analysis was carried out to determine the IC_50_ and the apparent K_i_ was calculated using the Cheng-Prusoff equation.

To explore this region in greater detail, six peptides that encompass most of the F2 sequence were generated (Table S1). Four of these peptides (F2P1, F2P2, F2P3, and F2P8), when included at increasing concentrations, inhibited prothrombin activation in a dose-dependent manner, while two of the peptides (F2P6 and F2P7) did not (Fig. S1). The four peptides that showed dose-dependent effects were then further investigated for their ability to inhibit prothrombin activation in a FVa-dependent manner. In the absence of FVa, F2P1 and F2P8 inhibited prothrombin activation while no effects were observed for F2P2 and F2P3 (Fig. 3A). Therefore, F2P1 and F2P8 were considered not to be involved in binding FVa and thus omitted in subsequent studies. In the presence of FVa (*i*.*e*. prothrombinase), F2P2 and F2P3, which also contain sequence overlaps with the longer peptide F2P-(16-36), inhibited prothrombin activation with estimated K_i_ values of 520 ± 92 μM and 31 ± 6 μM, respectively (Fig. 3B). Linear transformation using the Lineweaver-Burke plots showed a non-competitive type inhibition with comparable K_i_ values of 427 ± 4 μM and 34 ± 4 μM for F2P2 and F2P3, respectively (Fig. S2). Similar K_i_ values were also observed for these interactions in equilibrium binding using light scattering (Fig. S3). F2P2 and F2P3, respectively, showed 5- and 2-fold inhibition of prothrombin-FVa binding at saturation, which is noteworthy considering the multiple contacts engaged by prothrombin for binding FVa in prothrombinase^5^.

### Prothrombin activation monitored using DAPA

**Figure 4:**
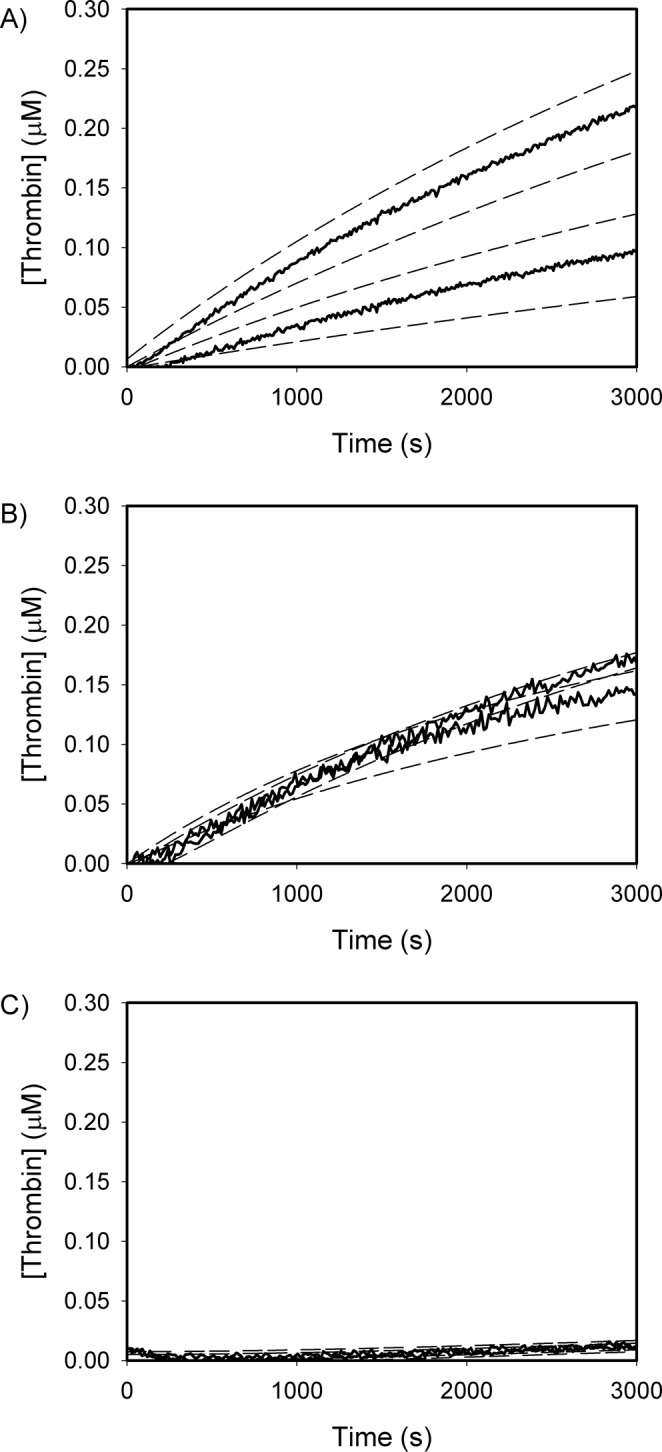
Thrombin generation profile of wild-type prothrombin and PT6 (1 μM) activation by (**A**) FXa/FVa, (**B**) FXa/PCPS, or (**C**) FXa alone, measured by FRET analysis using DAPA. Reaction mixtures for each set of conditions were as follows: (**A**) FXa (5 nM) and FVa (20 nM) (FXa/FVa), (**B**) FXa (5 nM) and PCPS (50 μM) (FXa/PCPS), and (**C**) FXa alone (5 nM). Under all conditions, experiments were initiated with the addition of FXa in HBST buffer with CaCl_2_ (5 mM) and DAPA (10 μM). Fluorescence was measured by a SpectraMax M2 fluorescent plate reader, with an excitation wavelength of 280 nm, emission wavelength of 545 nm and an emission cutoff at 530 nm at 20 s intervals.

**Figure 5:**
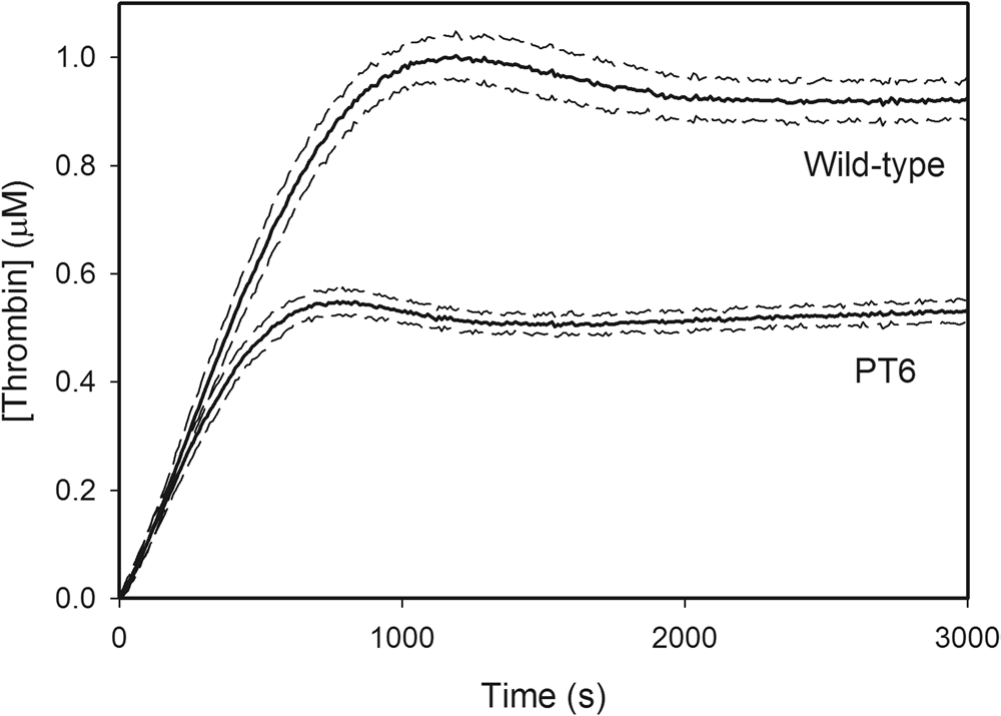
Thrombin generation profile of wild-type prothrombin and PT6 (1 μM) activation by the prothrombinase complex, measured by FRET analysis using DAPA. This reaction mixture contained FXa (20 pM), FVa (20 nM) and PCPS (50 μM). Under all conditions, experiments were initiated with the addition of FXa in HBST buffer with CaCl_2_ (5 mM) and DAPA (10 μM). Fluorescence was measured by a SpectraMax M2 fluorescent plate reader, with an excitation wavelength of 280 nm, emission wavelength of 545 nm and an emission cutoff at 530 nm at 20 s intervals.

The six residues that comprise the potential FVa binding site on F2 were then mutated (Gln177Ala, Gln179Ala, Arg181Ala, Leu182Thr, Val184Thr and Thr185Ala, in prothrombin numbering) creating a prothrombin mutant we refer to as PT6 (see experimental procedures for further detail). The functional consequences of these mutations were quantified and compared with the wild-type prothrombin using DAPA as the thrombin-specific probe^34^. The activation time course of prothrombin was used to measure the initial rates of thrombin generation by various combinations of prothrombinase components (Table 1). Significant differences in thrombin generation between the wild-type prothrombin and PT6 were observed only when FVa was present in the activation complex. When activated by FXa/FVa (Fig. 4A), the initial rates of thrombin generation measured from wild-type or PT6 prothrombin were (1.8 ± 0.5) × 10^−2^ s^−1^ and (5.3 ± 3.7) × 10^−3^ s^−1^, respectively; PT6 activation rate being 29% of that observed with the wild-type prothrombin. The reactions, however, were not complete even after 4 h, indicating that the overall thrombin generation is slow without the membrane surface. Total thrombin generated from PT6 was 35% of that observed with wild-type prothrombin, suggesting that the ability to generate mature thrombin is affected by the loss of these key residues within the F2 domain of prothrombin. With FXa/PCPS as the activator (Fig. 4B), the initial rates of activation were not significantly different between wild-type prothrombin (1.8 ± 0.5 × 10^−2^ s^−1^) and PT6 (1.4 ± 0.1 × 10^−2^ s^−1^). Similarly, with FXa alone (Fig. 4C), there were no significant differences between wild-type prothrombin (3.9 ± 4.3 × 10^−4^ s^−1^) and PT6 (5.1 ± 5.8 × 10^−4^ s^−1^). The differences in total thrombin levels generated between the two prothrombin variants were also minimal with either FXa/PCPS or FXa alone as the activator. With full prothrombinase (*i*.*e*. FXa/FVa/PCPS/Ca^2+^), a statistically significant difference in the initial rates of wild-type prothrombin (66.6 ± 9.6 s^−1^) or PT6 (55.1 ± 8.7 s^−1^) activation was observed (p < 0.05). Total thrombin generated was 42% lower for PT6 than wild-type prothrombin (Fig. 5). Taken together, these data suggest that the differences in activation between wild-type and PT6 prothrombin are apparent only in the presence of FVa.

**Table 1:** Initial rates of wild-type or PT6 prothrombin by FXa with varying components of prothrombinase.

	Wild-type		PT6		
	Rate	Fold	Rate	Fold	*p*
	*s* ^− *1*^		*s* ^− *1*^		
FXa	3.9 ± 4.3 × 10^−4^	1	5.1 ± 5.8 × 10^−4^	1	0.08
FXa, PCPS	1.8 ± 0.5 × 10^−2^	44.9	1.4 ± 0.1 × 10^−2^	27.3	0.29
FXa, FVa	1.8 ± 0.5 × 10^−2^	46.9	5.3 ± 3.7 × 10^−3^	10.4	<0.005
FXa, PCPS, FVa	66.6 ± 9.6	1.7 × 10^5^	55.1 ± 8.7	1.1 × 10^5^	<0.05

Wild-type or PT6 was activated by prothrombinase, FXa/FVa, FXa/PCPS, or FXa alone (see methods for concentrations). All reactions contain 5 mM CaCl_2_. Fold differences in initial rates are calculated separately for wild-type and PT6, relative to activation by FXa alone. Statistical significance was measured between wild-type and PT6 for each set of experiments using Student’s t-test. Values represent mean ± SD of at least two separate experiments in duplicates.

### Effects of selected residue mutations on the binding of prothrombin to FVa

**Figure 6:**
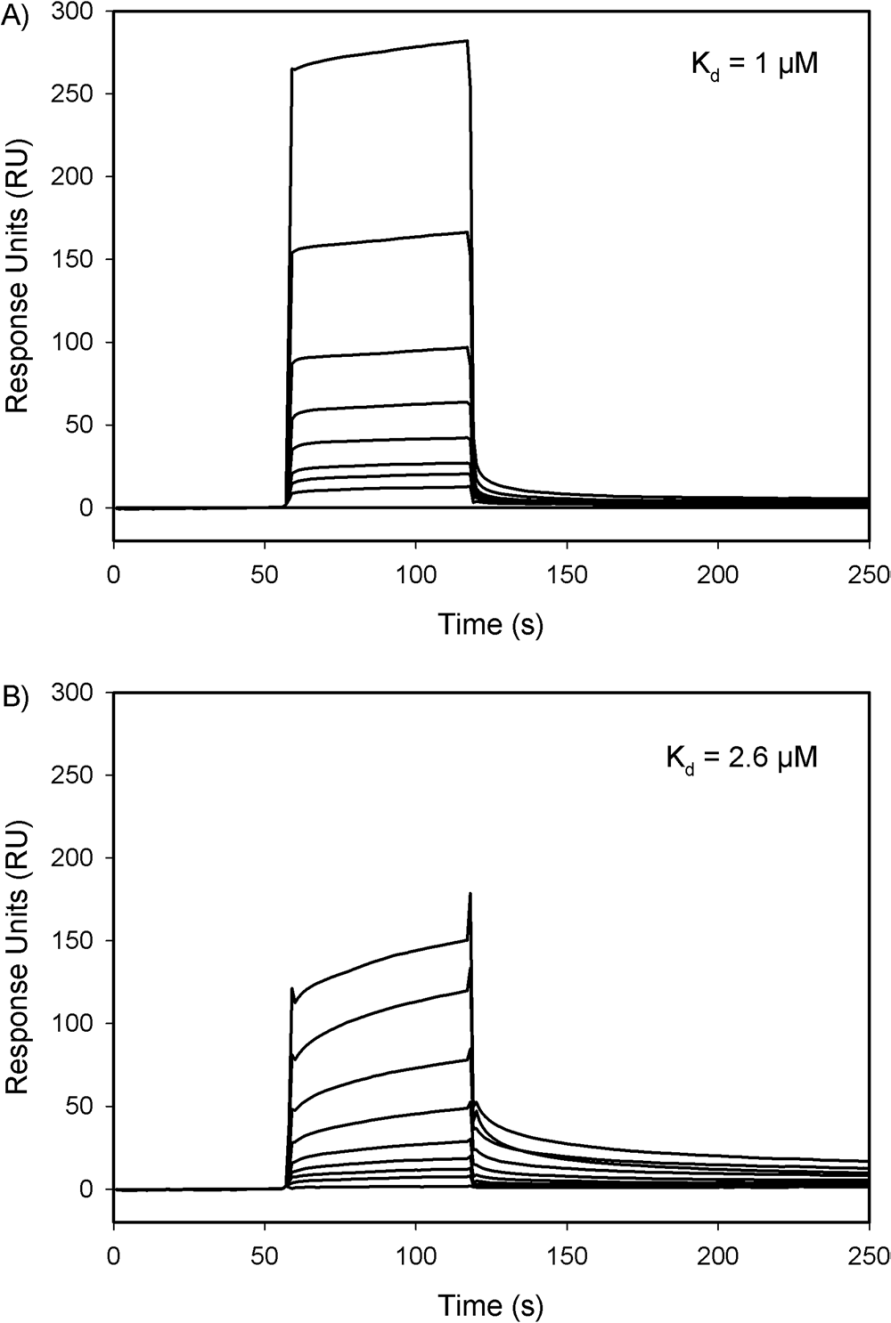
Binding of (**A**) wild-type or (**B**) PT6 prothrombin to FVa using surface plasmon resonance. Wild-type (**A**) or PT6 (**B**) prothrombin at varying concentrations (0, 78, 156, 313, 625, 1250, 2500, 5000 and 10,000 nM) were injected into a flow cell containing immobilized FVa for 60 s prior to washing with HBSC Tween-80. K_d_ values were determined using a kinetic 1:1 model.

The binding of wild-type prothrombin or PT6 to FVa was monitored in real-time and quantified using surface plasmon resonance (SPR). With FVa coupled onto a CM5 sensor chip, wild-type prothrombin showed a rapid association that was saturable, followed by a rapid and complete dissociation (Fig. 6A). PT6 demonstrated slower association, followed by slow dissociation (Fig. 6B). The maximum response unit (RU) change observed at 60 s was, on average, 33% less in PT6 compared with wild-type prothrombin of equal concentration. Kinetic rate analyses were used to estimate the affinity between prothrombin and FVa, as PT6 did not reach saturation and was not suitable for steady state analyses. The dissociation constant (K_d_) for PT6 (K_d_ = 2.6 μM) was 2.6-fold higher than wild-type prothrombin (K_d_ = 1 μM). Taken together, these data demonstrate that binding of prothrombin to FVa clearly involves the six residues identified within the F2 domain of prothrombin.

## Discussion

To date, the functional role of F2 in the prothrombin-FVa interaction has mostly been studied by the use of prothrombin variants with or without the F2 domain, and quantifying its activation by FXa/FVa. In particular, inclusion of isolated F2 was found to accelerate the FVa-dependent activation of prethrombin-2 both in the absence^35,36^ and presence^37,38^ of phospholipid membranes. One other study reported that F2 inhibits the cleavage of the full-length prothrombin by the FXa/FVa complex and that isolated F2 appears to have a comparable affinity of binding to FVa as that of prothrombin^39^. Binding quantified by NMR reveals specific interactions between the FVa-HC and residues located in the kringle portion of F2 (Fig. 2). Additional NMR studies also show the involvement of residues Asp223–Lys236 and the C-terminal peptide region of F2 in binding to thrombin^32^, which is in full agreement with the structural characteristics of the F2-thrombin complex formed non-covalently^27,28^ or covalently^40^. The FVa- and thrombin-binding sites on F2 are also spatially distinct from a sequence locus of F2 identified for binding FXa^13,14^. In all, residues of F2 perturbed by binding of the FVa-HC are different from those responsible for interactions with thrombin, which suggests that the same residues of the F2 domain of prothrombin and F2-containing zymogen intermediates, such as prethrombin-1^9,35^ or the functionally equivalent F2-prethrombin-2 complex^27,28,38,41^ should also be available for binding FVa.

While the difference in initial rates of prothrombin activation by prothrombinase between wild-type and PT6 were minimal (Table 1), there was a large difference in total thrombin generation at completion (Figs 4 and 5), comparable with the effect observed in the peptide inhibition studies. A similar observation has been reported previously by our group^42^, whereby decreases in total thrombin generation are not accompanied by proportional decreases in the initial rates of prothrombin activation. Effects on the initial rates of thrombin generation, as well as total thrombin generation were accentuated only when prothrombin was activated in the presence of FVa, indicating FVa dependence of inhibition. This is consistent with the results obtained using SPR whereby the loss of key residues within the F2 region significantly weakened the binding of prothrombin to FVa.

The main function of FVa in the prothrombinase complex is to promote the efficient conversion of prothrombin into thrombin^25^, in part by mediating the interaction of a mature exosite I in meizothrombin^43^ with a negatively-charged C-terminal region of the FVa-HC^44–46^. Binding between prothrombin and FVa is also thought to involve the F2 domain^26,37,47^ and the F1 domain^10,39^. Such binding interactions of prothrombin with FVa involving multiple contact sites are normally interpreted^10^ as having complete tertiary structural assembly between multi-domain proteins^48,49^. However, the initial interaction between FVa-HC and prothrombin appears to be partially dispensable. Thrombin generation remains largely unaffected when prothrombinase is composed of truncated forms of FVa lacking portions of the C-terminal tails of the heavy chain^44,46^. As well, potently inhibiting peptides derived from the C-terminus of the FVa-HC only partially reduced the enhanced thrombin generation by the FXa/FVa complex, compared with membrane-bound FXa alone^44,50^. Taken together, this suggests the functional interaction between FVa and prothrombin can also engage a subset of all possible contact sites within prothrombin, including proexosite I, Gla- and kringle 1 domain of F1, and F2.

It has been widely accepted that FVa alone increases the rate of thrombin generation by 3 orders of magnitude while the membrane surface alone increases the catalytic efficiency of thrombin generation by 2 orders of magnitude, which together increases its catalytic efficiency by 5 orders of magnitude^5^. These individual contributions of the prothrombinase components toward overall thrombin generation, however, were not observed in our experiments (Table 1). The difference in rate enhancement of thrombin generation afforded by FVa or PCPS alone was similar (~45-fold). The 5-orders of magnitude enhancement as reported in literature was only observed upon the full assembly of prothrombinase. A similar trend has previously been reported during FX activation by the intrinsic tenase complex (FIXa/FVIIIa/membrane surface), whereby addition of either the cofactor FVIIIa or the membrane surface each results in the enhancement of tenase by 2 orders of magnitude, but assembly of the full tenase complex enhances its activity by 6 orders of magnitude^6^. These findings demonstrate that the effect of individual components of multivalent enzymatic complexes do not always show simple multiplicative effects of independent binding events. The overall reaction rate is reflective of all binding modes between the enzyme, cofactor, lipid surface, and the substrate that must all work in concert for maximal efficiency of a fully-assembled catalytic complex. This idea has also been shown in the context of the prothrombinase complex, where prothrombin variants lacking membrane binding potential (desGla) showed very modest decreases in the rate of thrombin generation (5-fold)^51^, compared with the 2-orders of magnitude decrease upon loss of membrane binding historically described in literature. Interestingly, desGla prothrombin was shown to be more efficiently cleaved at Arg271 than wild-type, demonstrating that alternate binding modes were being utilized in processing prothrombin by prothrombinase. This information is in agreement with our new results and rationalizes the overall enhancement that is greater than the multiplicative of the individual contributions provided by each prothrombinase component during thrombin generation.

Multivalent molecular complexes can engage in partial/dynamic binding modes, and has been observed with polyvalent biopolymers^15^, prothrombinase^19,20^, and other coagulation complexes^21^. These partial binding modes would allow for thrombin generation to occur, even without a complete association of the enzyme-substrate complex, a phenomenon quite prevalent in the enzymatic processing of polymeric or multi-domain substrates^20,21,52^. Such intrinsic heterogeneity of multivalent prothrombin-FVa interactions may explain the reported weak inhibitory capacities of the FVa-binding Gla- and kringle 1 domains of prothrombin, despite their high affinities for FVa^10,39^. This may also explain the partial inhibitory effect observed for a monoclonal antibody specific for the F2 domain of prothrombin^26,47^. As well, a peptide derived from residues 55–65 of hirudin was found to have much higher inhibitory effect on prothrombin activation by the FXa/FVa complex in the absence of a membrane surface^53–55^. Partial assembly of multivalent molecular complexes provides opportunity for accelerated rate of complex dissociation^15^. This may be advantageous for modulating its activity rather than being dependent on inhibition by a specific inhibitor. Multivalent complexes also offer the substrate (*i*.*e*. prothrombin) to have a greater contribution in its activation mechanism^20,22,23^ and opportunities for dynamic control of thrombin generation under physiological requirements other than blood coagulation^22–24^. Together with our data that identify residues of prothrombin F2 that interact with FVa, we therefore demonstrate that the overall enhancement of thrombin generation afforded by full prothrombinase complex formation is consistent with other reported multivalent molecular complexes whereby all interactions, including the substrate, can work in concert to provide the overall mechanism and ultimate efficiency of substrate processing.

## Experimental Procedures

### Materials

Various materials and their sources are listed in the Supplementary Materials.

### Synthetic Peptides

Peptides were synthesized by the solid-phase method using standard FMOC chemistry on an Applied Biosystems 431A peptide synthesizer (Sheldon Biotechnology Center, McGill University). The details are outlined in Supplementary Materials.

### Preparation of FVa heavy chain and bovine thrombin

The FVa heavy chain for NMR studies was prepared from purified bovine FVa, following a procedure as described previously^45^. Briefly, FV was activated to FVa by the addition of thrombin (1.96 units/mL) at 37 °C for 25 min and stopped with Phe-Pro-Arg-chloromethylketone (FPRck, 1 μM). The heavy and light chains of FVa were isolated using immunoaffinity column chromatography as described previously^56^. The chains were then pooled separately, precipitated against 80% ammonium sulfate, and stored at 4 °C.

### Molecular cloning and protein expression with Pichia pastoris

Recombinant expression of human prothrombin F2 encompassing residues 156–271 of prothrombin was performed in the yeast *Pichia pastoris* as described previously^32^. Briefly, the cDNA encoding the sequence of F2 was amplified by PCR using a subcloned segment of the prothrombin cDNA as a template. cDNA encoding F2 was inserted into the pPIC9 expression vector, whereby the final F2 expressed contains six-histidine tags in its C-terminus separated by the natural FXa cleavage site. The expressed F2 protein was first purified by nickel-agarose chromatography and then treated by FXa to remove the His-tag. After removing FXa using benzamidine Sepharose, purity was assessed by SDS-PAGE and its molecular mass determined (12,605 Da) using both the ESI (Perkin-Elmer Sciex) and MALDI-TOF (Kratos Analytical) methods.

### NMR sample preparation

Uniform ^15^N and ^13^C/^15^N isotopic labeling of F2 was achieved as described previously^32^. Once labeled, F2 preparations prepared and pH adjusted, 25 μL of D_2_O was added to the protein solution to provide the NMR deuterium lock signal. For the titration experiments, small aliquots of the FVa-HC or FPRck-thrombin, both in 2 mM HEPES, 50 mM sodium phosphate buffer, pH 7, were added to samples of ^15^N-labeled F2 up to a molar ratio of ~10:1. A thrombin-derived peptide (see Synthetic Peptides in Supplementary Materials) (in 2 mM HEPES, 50 mM sodium phosphate buffer at pH 7) was also added to the ^15^N-labeled F2 to a molar ratio of 1:3 with the thrombin peptide in molar excess. ^1^H-^15^N HSQC experiments were utilized to follow binding interactions of F2 with the addition of FVa-HC, thrombin and the thrombin-derived peptide.

### NMR experiments

All NMR experiments were carried out at 35° C on a Bruker Avance500 or an Avance800 spectrometer equipped with triple-resonance (^1^H, ^13^C, and ^15^N) and three-axis gradient probes. Two-dimensional and three-dimensional NMR data collected for ^15^N- and ^13^C/^15^N-labeled F2 included ^1^H-^15^N HSQC, HNCO, HN(CA)CO, HNCA, HN(CO)CA, CBCA(CO)NH and HNCACB^57–59^. Water suppression was achieved using the WATERGATE method with a 3:9:19 selective pulse incorporated in all the three-dimensional pulse sequences^60^. To improve water suppression, water magnetization was re-aligned to the +*Z*-axis before acquisition by setting the phase of the last 90° ^1^H pulse to −*X*. For the HNCA experiment, SEDUCE-1 decoupling with a field of 2.5 kHz^61^ was used to decouple ^13^CO-^15^N interactions in the *t*1 and *t*2 evolution periods. All data sets were processed using NMRPipe^62^ with 90°-shifted sine-square weighting functions in all three dimensions. Spectral display and initial assignments were carried out using the XEASY software package^63^. Sequence-specific assignments of the backbone (HN, ^15^N, ^13^C_α_ and some ^13^C_β_) resonances of human F2 perturbed by FVa were achieved by use of a combined analysis of the two-dimensional TOCSY/NOESY and three-dimensional HNCO, HN(CA)CO, HNCA, HN(CO)CA, CBCA(CO)NH and HNCACB experiments.

### Effects of F2 and peptides on prethrombin-2 and prothrombin activation

Recombinant and plasma-derived F2 were characterized through the activation of prethrombin-2 (100 nM) by prothrombinase (FXa at 50 pM, FVa at 100 pM, PCPS vesicles at 10 μM and calcium ion at 2.5 mM in 20 mM Tris-HCl, 0.15 M NaCl, pH 7.4 (TBS) at 37 °C in the absence or presence of F2 (200 nM). Activation of prothrombin (300 nM) was carried out in the presence of PCPS (50 μM), FVa (5 nM), and DAPA (5 μM) and various concentrations of each peptide in TBS with 5 mM CaCl_2_ and 0.01% Tween-80. Reactions were carried out in 96-well plates which had been pre-treated with TBS with 1% Tween-80 and rinsed thoroughly with water. Reactions were started with addition of FXa (0.1 nM), and monitored in SpectraMax Gemini fluorescence plate reader (Molecular Devices, Sunnyvale, California) at excitation and emission wavelengths of 280 nm and 545 nm, respectively, with a 530 nm emission cut off filter in the emission beam at 25 °C. In the absence of FVa, reactions were carried out as described above; however, the reactions were started with the addition of 20 nM FXa. The K_i_ values were estimated using the Cheng-Prusoff equation (IC_50_ = [1 + ([S]/K_M_)] K_i_) based on the IC_50_ values obtained by nonlinear regression of a rectangular hyperbola, whereby [S] is the prothrombin concentration and K_M_ is the Michaelis constant of prothrombinase for prothrombin determined in a separate experiment as described previously^19^.

### Affinities of prothrombin variants for FVa determined using SPR

Binding interactions were studied by SPR as described previously^64,65^ using a Biacore T200 (GE Healthcare). Briefly, FVa was immobilized on a CM5 sensor chip (GE Healthcare) using an amine coupling kit (GE Healthcare). After injecting the 1-ethyl-3-(3-dimethylaminopropyl)-carbodiimide hydrochloride/N-hydroxysuccinimide mixture into the flow cell at a rate of 10 μL/min for 420 s, 28 μg/mL FVa in 10 mM acetate buffer, pH 5.5 was injected at a rate of 5 μL/min until 8000–9000 response units (RU) were immobilized. Flow cells were then washed with 1 M ethanolamine for 420 s, followed by 0.02 M HEPES, 0.15 M NaCl, pH 7.4 (HBS) with 5 mM CaCl_2_ and 0.01% Tween-80.

To measure the binding of prothrombin to FVa, increasing concentrations (0, 78, 156, 313, 625, 1250, 2500, 5000 and 10,000 nM) of wild-type or PT6 prothrombin were injected into the flow cells at a flow rate of 30 μL/min for 60 s, followed by buffer for 300 s. Flow cells were regenerated with 500 mM CaCl_2_ between sample injections. K_d_ values were calculated by estimating the individual on- and off-rate constants based on fitting of the sensorgrams to 1:1 Langmuir binding model using BIAevaluation software (GE Healthcare). Samples were analyzed twice in duplicate.

### Generation and isolation of a prothrombin derivative, PT6

Six out of the seven residues of F2 within a 9-residue cluster identified by heteronuclear NMR were altered by site-directed mutagenesis (Gln177Ala, Gln179Ala, Arg181Ala, Leu182Thr, Val184Thr and Thr185Ala; prothrombin numbering). using the QuikChange Lightening Site-Directed Mutagenesis kit as described previously^66^. Mutations of the six residues were introduced in two-steps, with three mutations per reaction, using primers 5′-GATCGGGGGCAGGCGTACGCGGGGGCCCTGGCGGTGACCACAC-3′ and 5′-GCGTACGCGGGGGCCACGGCGACGGCCACACATGGGCTCCCC-3′. DNA was then isolated using the QIAprep Spin Miniprep Kit and was sequenced and amplified using Plasmid Maxi Kit. All DNA fragments were sequenced at Robarts Research Institute (London, Canada). PT6 cDNA was then transfected into baby hamster kidney cells, and the expressed PT6 was isolated as described previously^19^. The purified PT6 was then subjected to precipitation by 80% ammonium sulfate and stored at −20 °C in 50% glycerol.

### Thrombin generation measured by DAPA fluorescence

Prothrombin activation was quantified using DAPA as described previously^34^. Wild-type or PT6 prothrombin (1 μM) was diluted into HBS with 0.01% Tween-80 and 0.1% Prionex (Pentapharm, Switzerland), and subsequently mixed with CaCl_2_ (5 mM), DAPA (10 μM) and varying mixtures of FVa and PCPS. To initiate the reaction, these mixtures were then transferred to FXa-containing wells in a 96-well microtitre plate. Experiments using full prothrombinase included PCPS (50 μM), FVa (20 nM) and FXa (20 pM). The incomplete prothrombinase compositions were: (1) FXa (5 nM) and FVa (20 nM) (FXa/FVa), (2) FXa (5 nM) and PCPS (50 μM) (FXa/PCPS), and (3) FXa alone (5 nM). Fluorescence was measured by a SpectraMax M2 fluorescent plate reader, with an excitation wavelength of 280 nm, emission wavelength of 545 nm and an emission cutoff at 530 nm at 20 s intervals. The fluorescence quantum yield of DAPA-thrombin complex was determined experimentally to be 2065.9 RFUs/μM thrombin.

## Supplementary information

Supplementary Materials
