# Supplementary material for: Identification and characterization of a factor Va-binding site on human prothrombin fragment 2
Source: Sci Rep. 2019 Feb 21;9:2436. doi: 10.1038/s41598-019-38857-4 (PMC6385242; doi:10.1038/s41598-019-38857-4)
Supplement: Supplementary file 1 — Supplementary Materials [file 41598_2019_38857_MOESM1_ESM.pdf]

Supplementary material for the manuscript:

Identification and characterization of a factor Va-binding site on human prothrombin fragment 2

Alexander P. Friedmann, Anatoli Koutychenko, Chengliang Wu, James C. Fredenburgh, Jeffrey I. Weitz, Peter L. Gross, Ping Xu, Feng Ni, Paul Y. Kim

Methods

Materials – Human FXa and FVa were purchased from Haematologic Technologies, Inc (Essex Junction, VT, USA). Human plasma-derived wild-type prothrombin and the F2 domain protein derived from human prothrombin were purchased from Enzyme Research Laboratories (South Bend, IN, USA). Bovine plasma barium citrate eluate and dried venom of the snake *Echis carinatus*, phosphatidyl-L-serine (PS), phosphatidyl-L-choline (PC), and XAD-2 resin were purchased from Sigma. Bovine FVa was purified from bovine plasma as described previously (1). The anti-FVa light chain-mouse monoclonal antibody E9 was a generous gift from Drs. Mann, Orfeo, and Brummel-Ziedins (University of Vermont, USA). QuikChange Lightening Site-Directed Mutagenesis Kit was purchased from Agilent Technologies (Santa Clara, CA, USA). DNA primers were synthesized by Integrated DNA Technologies (Coralville, IA, USA). QIAprep Spin Miniprep Kit and Plasmid Maxi Kit were purchased from Qiagen (Hilden, Germany). Lipofectamine 3000 Transfection Kit was purchased from Life Technologies-Invitrogen (Carlsbad, CA, USA). Baby hamster kidney cells and the pNUT vector, used for mammalian expression, were provided by Dr. Ross MacGillivray (University of British Columbia). Methotrexate (Mayne Pharma Inc., Montreal, Quebec Canada) and Vitamin K1 were purchased from Hamilton General Hospital. Q-Sepharose Fast Flow anion-exchange resin and Mono-Q HR 5/5 column were obtained from GE Healthcare (Burlington, Ontario Canada). Gibco® D-MEM/F-12 media, newborn calf serum and Opti-MEM I media, 0.5% Trypsin-EDTA and Antibiotic-Antimycotic solution were purchased from Thermo Fisher Scientific (Carlsbad, CA, USA). Matched-Pair Antibody Set for ELISA of Human Prothrombin Antigen, was purchased from Affinity Biologicals Inc (Ancaster, ON, Canada). The fluorescent α-thrombin inhibitor dansylarginine-N-(3-ethyl-1,5-pentanediyl)amide (DAPA) was prepared as previously described (2). Phospholipid vesicles composed of 75% PC and 25% PS (PCPS) were prepared as previously described (3). Bio-Rad Protein Assay reagent was purchased from Bio-Rad (Mississauga, ON, Canada).

Synthetic Peptides – Peptides were synthesized by the solid-phase method using standard FMOC chemistry on an Applied Biosystems 431A peptide synthesizer (Sheldon Biotechnology Center, McGill University). A peptide encompassing residues 91-101 in the B-chain of human thrombin (4) was prepared with two Cys residues added, one before position 91 and one after position 101 to produce a cyclic molecule $CH_{91}PRYNWRENLDR_{101}C$. One step free-air oxidation of the peptide was performed overnight in 1% ammonium acetate buffer, pH 8.5, at a peptide concentration of ~10 μg/ml. The oxidized peptide was purified on a Vydac C18 HPLC column with a linear 10-45% gradient of acetonitrile in an aqueous solution containing 0.1% TFA. Peptides that encompass F2 were prepared with the sequences outlined in Table S1. F2 sequence corresponding to prothrombin residues 218-231 was ignored, as previous studies using a monoclonal antibody directed toward this sequence eliminated it as a potential binding site for

FVa (5). All peptides were weighed and solubilized in a measured volume of water, excluding F2P4 and F2P5, which were not soluble in water, 0.02 M Tris-HCl, 0.15 M NaCl, pH 7.4 (TBS), or DMSO. Therefore, F2P4 and F2P5 were excluded from the study. Ion-spray mass spectrometry (SIEX API *III*) was used to confirm the identity of the purified peptide.

Light scattering equilibrium binding studies – 90º light scattering was measured using LS50B fluorescence spectrophotometer (PerkinElmer Life Sciences), and the reactions were carried out in a quartz cuvette containing TBS with 5 mM $CaCl_2$ and FV (1 μM). The signal was monitored at an excitation and emission wavelengths of 320 nm. Thrombin (2.5 nM) was added to activate FV to FVa. FPRck (25 nM) was added to inhibit the thrombin. Prothrombin (10 μM) was added, followed by increasing concentrations of F2P2 or F2P3. The signal was allowed to stabilize between each addition. Control experiments containing peptide only, and peptide with FVa only were carried out in each case. Utilizing the K_d for FVa–prothrombin binding of 10 μM (6), we were able to calculate the % change expected in scattering intensity if the peptides were able to inhibit this interaction. In both cases we expected that upon addition of prothrombin, approximately 10% of the total increase in scattering intensity was due to prothrombin-FVa interaction. Therefore, we would expect the appropriate 10% decrease in the signal upon perturbation of this interaction. The apparent K_i value was calculated from the IC_{50} value for peptide inhibition of FVa-prothrombin interaction (7).

SDS-PAGE time-course analysis of prothrombin activation – Prothrombin (1.4 μM) in a mixture of PCPS (50 μM), FVa (20 nM), $CaCl_2$ (5 mM), DAPA (10 μM), buffered with 0.02 M Hepes, 0.15 M NaCl, pH 7.4, 0.01% Tween80 (HBST) with 0.1% prionex, was activated by 20 pM FXa. Samples were removed at respective time points, and reactions were stopped by adding samples into acetic acid at a final concentration of 0.134 N. Samples were dried using SpeedVac, reconstituted in sample buffer and loaded onto Bio-Rad Criterion TGX 8-16% gels. These gels were resolved, stained for at least 1 h with Bio-Rad Coomassie Brilliant Blue G-250 and destained for at least 30 min with deionized water prior to imaging.

Quantitative densitometry was carried out using Bio-Rad Image Lab (V5.2.1). Band intensities were summed for each well and normalized to the prothrombin band of lane 1 (1.4 μM). These values were then converted into concentration as described previously (8) using the correction coefficient for the respective fragment when using Coomassie Blue. Correction coefficients were 0.849, 0.4468, 0.3695, 0.6265 and 0.5492 for Pre1, F1.2-A chain (F1.2A), F1.2, Pre2 and B-chain, respectively. Calculated values were then plotted against reaction time to investigate the appearance of intermediates and products.

Results

Functional characterization of recombinant F2 – The identity and functional properties of recombinant F2 were examined by comparison with plasma-derived F2 in promoting the turnover of prethrombin-2 by prothrombinase (9). Recombinant F2 produced by *P. Pastoris* increased the rate of prethrombin-2 activation, in a fashion almost indistinguishable from the

plasma-derived material, indicating purity and functional integrity of the recombinant F2 protein (9).

Recombinant F2 also exhibited similar one-dimensional proton NMR spectra as natural F2 prepared from plasma prothrombin (9). Notably, there were two upfield-shifted methyl proton resonances, which are common for a conserved Leu residue in all kringle domains (10–12). Some backbone ^{1}H and ^{15}N resonances or HSQC spectrum of ^{15}N-labelled F2 were assigned and additional resonance assignments of sidechain β-carbons and β-protons indicated that the secondary structure of F2 in solution is in general agreement (9) with those in the crystal structure of the kringle 2 (K2) domain or residues Cys170-Cys248 of human prothrombin (4, 13). The N- and C-terminal regions of F2 outside of the kringle domain were found to have a certain tendency for helical conformations for residues 98-103 (or prothrombin residues 253-258) in the C-terminal region of F2 that was not defined in the X-ray structure of F2 in complex with thrombin (4, 13). These prothrombin residues (*i.e.* Glu253-258) appear as an inter-domain linker with extended conformations in both the crystal structures of meizothrombin-desF1 (pdb: 3E6P (14)) and prethrombin-1 (pdb: 4NZQ (15)). Interestingly, even the kringle (K2) region of F2 appears to have a substantial conformational flexibility, as exhibited by more pronounced differential broadening of many NMR (H,^{15}N)-HSQC peaks at a higher magnetic field strength (800 MHz) as compared to a lower field strength (500 MHz) (spectrum not shown).

As a comparison, HSQC spectra collected for ^{15}N-labeled F2 in the presence of thrombin with thrombin/F2 molar ratios of 1:30, 1:20, 1:15, and 1:10 contain perturbed peaks that can be separated into two major clusters; one comprising residues Asp68–Lys81 of the kringle domain of F2 and the second Ala96–Asp108 of the C-terminal peptide region (9).

X-ray structures of the F2-thrombin complexes defined a surface loop at the exosite II region of thrombin, residues 91-101, in structural contacts with a cavity formed by residues Asp68-Lys81 of the F2 domain. To further demonstrate the specificity of F2 binding interactions, we synthesized a peptide corresponding to residues 91-101 of human thrombin with two cysteine residues added to form a disulfide bond between the N- and C-terminal ends of the peptide (see Experimental Procedures). This peptide contains in particular three of the four Arg residues (Arg93, Arg97, Arg101 and Arg 175 of thrombin) that make strong structural contacts with the anionic DGDEE motif of F2. Potential binding of the thrombin-derived peptide with F2 was monitored by the ^{1}H-^{15}N HSQC spectra of ^{15}N-labeled F2 with a F2:peptide ratio of 3:1. The thrombin-derived peptide generally induced smaller chemical shift changes than those induced by intact thrombin (9); however, the same region of F2 was affected by both thrombin and the thrombin fragment, which are distinct from F2 residues perturbed by FVa binding (Fig. 2). Taken together, residue-specific resonance shifts in F2 by FVa heavy chain must represent genuine molecular contacts between F2 and FVa despite the apparently low-affinity binding that is manifested by progressive titration shifts.

Equilibrium binding of FVa–prothrombin in the presence of F2P2 and F2P3 – Activation of FV to FVa and subsequent interaction between prothrombin and FVa with increasing levels of the inhibitory peptides, F2P2 or F2P3, was monitored by 90º angle light scattering (Fig. S3). Initially upon addition of a 10-fold excess of prothrombin, the scattering intensity increased due to the presence of both free prothrombin and FVa-prothrombin complexes. As outlined in the Experimental Procedures, we calculated that 10% of the increase was due to FVa-prothrombin complexes and that inhibition of this interaction would lead to a 10% decrease in scattering

intensity. In both cases, a decrease in intensity was observed upon addition of F2P2 and F2P3 in a dose-dependent manner. F2P2 exhibited a total change in scattering intensity of approximately 26 units at saturation (approximately 18 units expected), and a calculated apparent K_i value of 387 ± 79 μM. F2P3 exhibited a total change in scattering intensity of approximately 50 units (approximately 20 units expected), with a calculated apparent K_i of 28 ± 5 μM. Both values are in good agreement with the K_i values derived from the kinetic experiments. These results indicate that F2P3 has a higher binding affinity for FVa than F2P2, but is a less potent inhibitor of prothrombinase catalytic activity towards prothrombin.

Time-courses of prothrombin consumption and intermediate/product formation – To visualize the potential differences in prothrombin consumption as well as intermediate and thrombin generation during activation of the WT prothrombin (WTPT) or the six-mutation prothrombin (PT6), SDS-PAGE was utilized to generate time course profiles of each species (Fig. S4). There were clear differences in the band intensities between WTPT and PT6, especially in meizothrombin, which is inferred by the F1.2-A-chain band. Quantitative densitometry was performed on these bands to determine the time courses of prothrombin, meizothrombin, F1.2, and the B-chain of thrombin (Fig. S5). Prothrombin cleavage (Fig. S5A) was lower in PT6 compared with WTPT. The total meizothrombin generation (Fig. S5B), which was quantified by calculating the area under the curves of each plot, was roughly 50% lower in PT6 than WTPT. Although the differences in the F1.2 time course was minimal (Fig. S5C), there was a decrease in B-chain accumulation for PT6 compared with WTPT (Fig. S5D).

Supplementary Figures

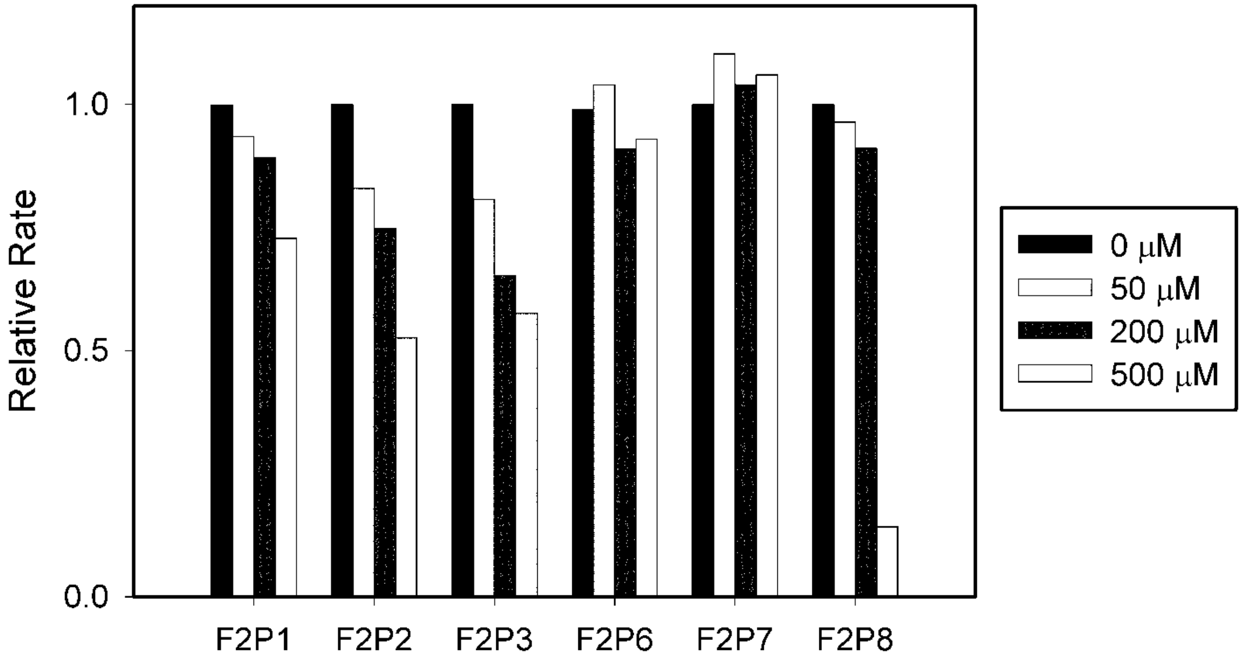

Figure S1. Relative rate of prothrombin activation in the presence of each of the peptides at various concentrations. Initial rates of prothrombin activation were determined from reactions containing 300 nM prothrombin. 5 mM $CaCl_2$, 3 μM DAPA, 50 μM PCPS, 5 nM FVa and 0.05 nM FXa. Relative rates of prothrombin activation are shown for each concentration of peptide at 0, 50, 200 and 500 μM, respectively.

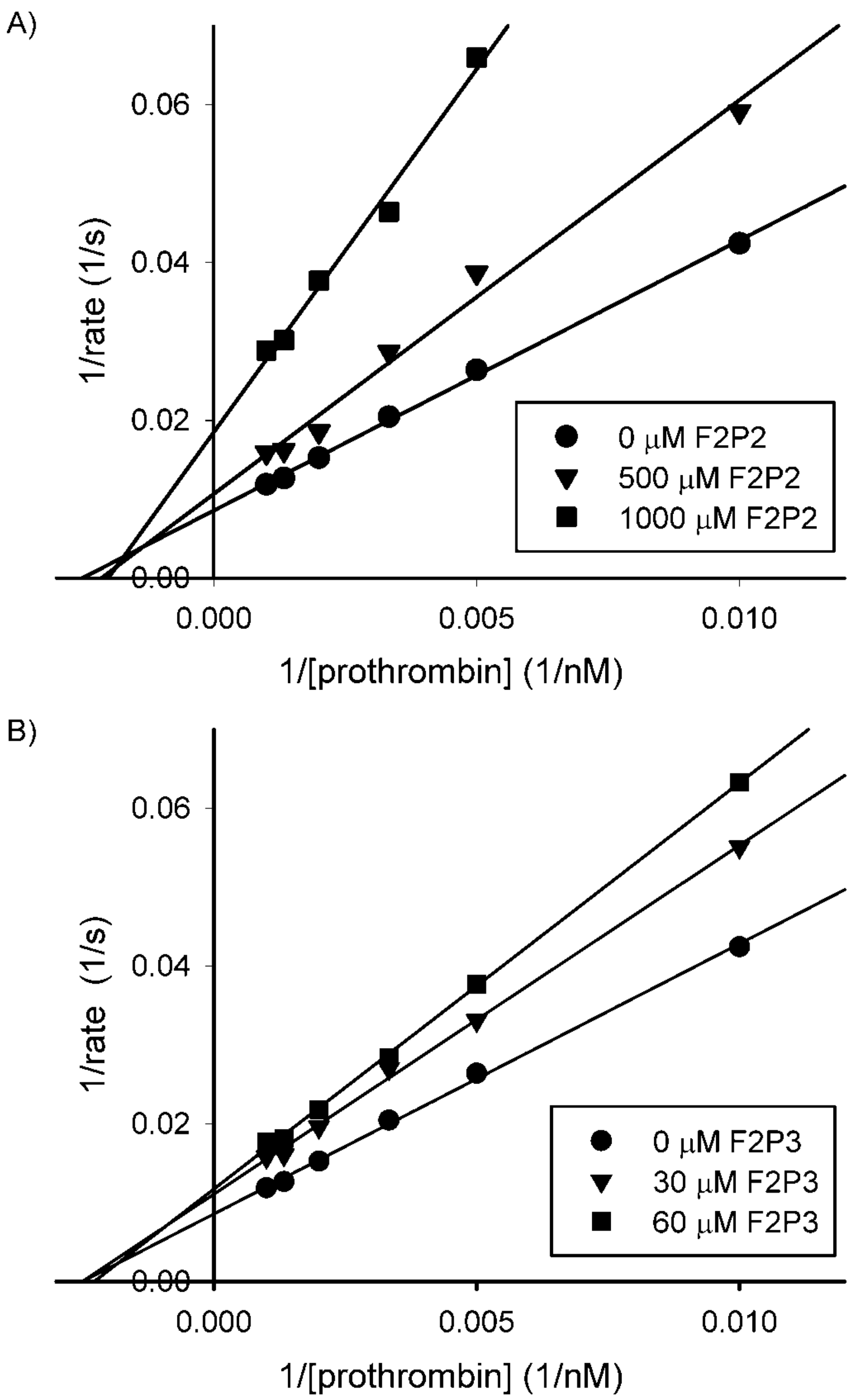

Figure S2. Lineweaver-Burke plots for prothrombin activation in the presence of F2P2 and F2P3. Initial rates of prothrombin activation were determined in a fluorescent plate reader, at room temperature, in the presence of 300 nM prothrombin, 5 mM $CaCl_2$, 5 μM DAPA, 50 μM PCPS, 5 nM FVa, and A) F2P2 or B) F2P3. Reactions were started by addition of 0.05 nM FXa.

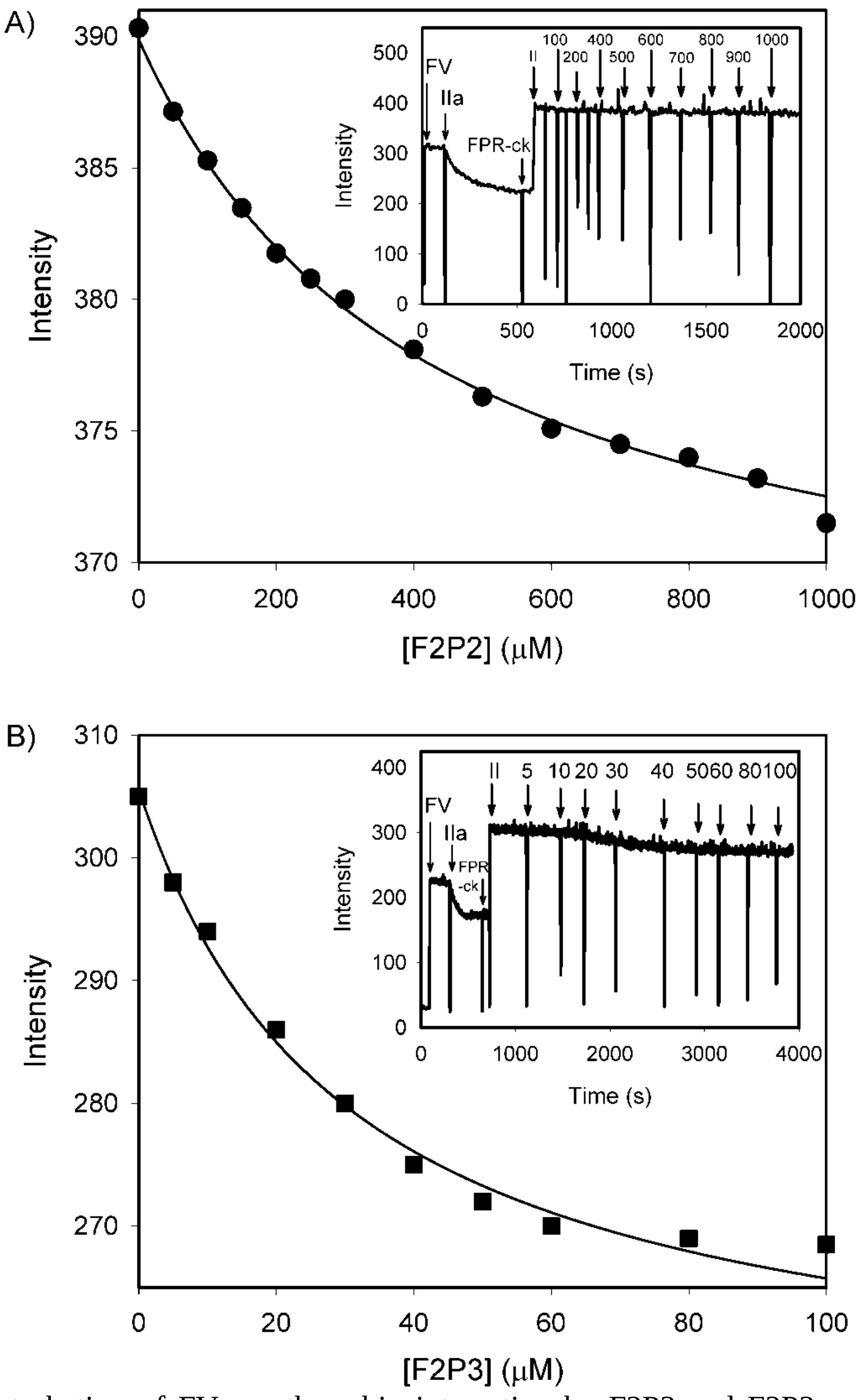

Figure S3. Perturbation of FVa-prothrombin interaction by F2P2 and F2P3 as determined by light scattering. 90° light scattering was measured in a quartz cuvette containing 0.02 M Tris-HCl, 0.15 M NaCl, 5 mM $CaCl_2$ and FV (1 μM), at room temperature. The signal was monitored at an excitation wavelength of 320 nM and an emission wavelength of 320 nM. Thrombin (2.5 nM) was added to activate FV to FVa. FPRck (25 nM) was added to inhibit the thrombin. Prothrombin (10 μM) was added, followed by increasing concentrations of A) F2P2 or B) F2P3. Raw data is inset in each case. The change in intensity was calculated upon peptide addition and plotted vs. peptide concentration. Non-linear regression analysis was used to calculate the apparent K_i values.

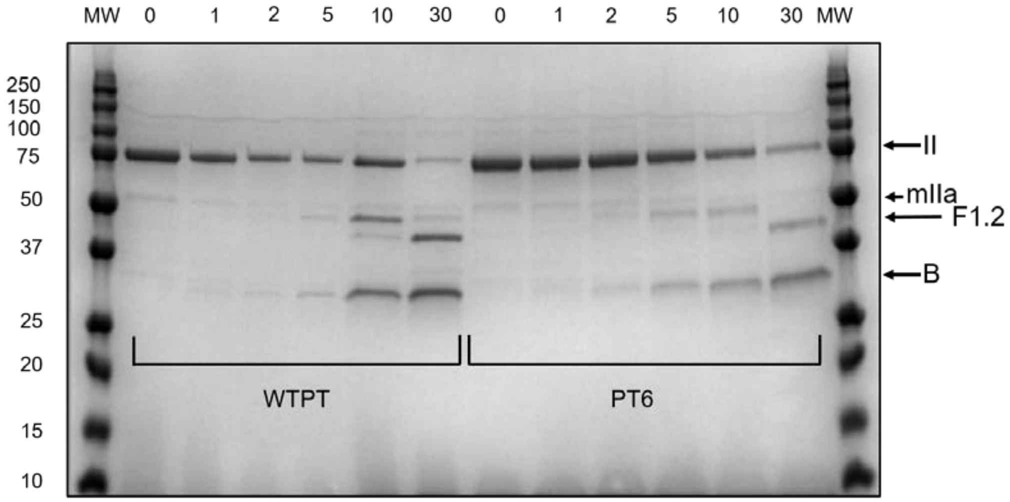

Figure S4. SDS-PAGE time-course analysis of prothrombin activation by prothrombinase. WTPT or PT6 (1.4 μM) was incubated with FVa (20 nM), PCPS (50 μM), $CaCl_2$ (5 mM), DAPA (10 μM) in HBST. Reactions were initiated by the addition of FXa (20 pM) at 25ºC. Samples were removed at indicated times by adding into 0.134 N acetic acid and the contents were resolved by SDS-PAGE under reducing conditions.

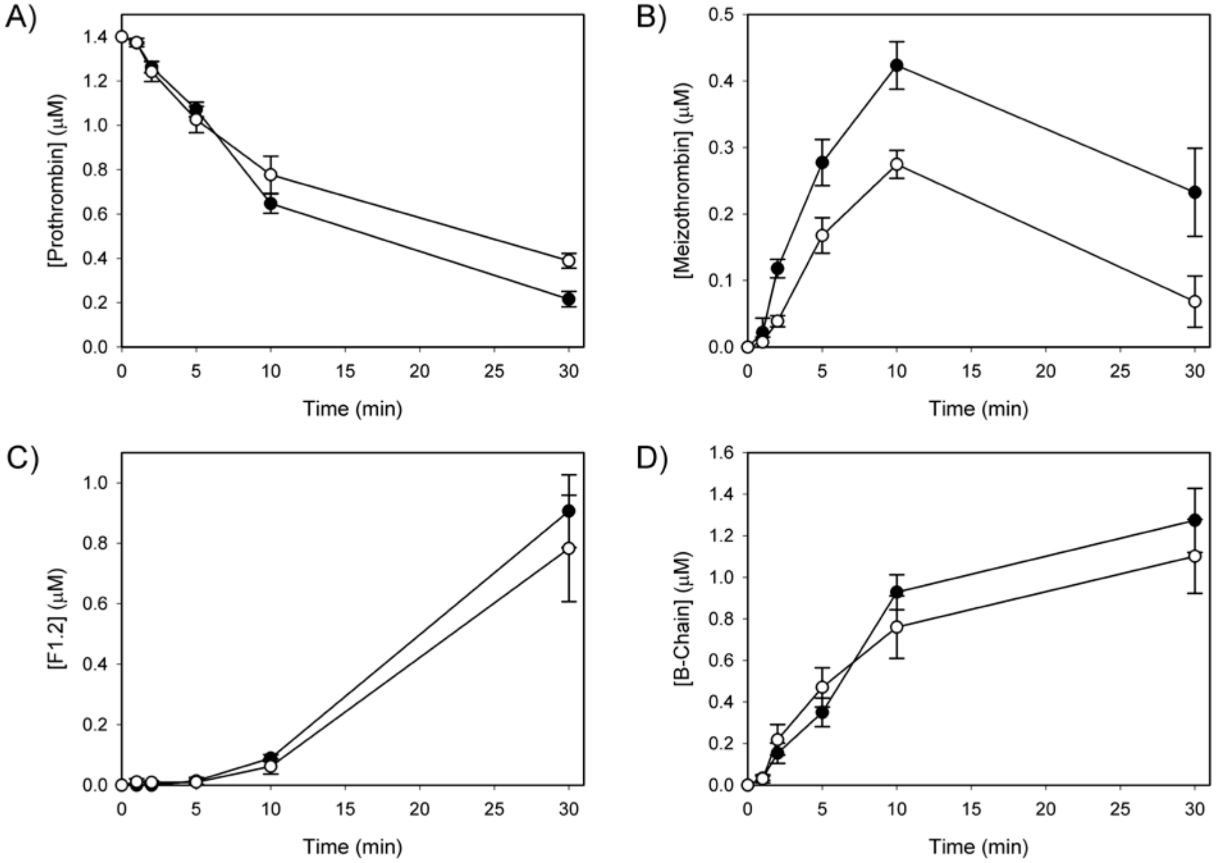

Figure S5. Quantitation of time-course activation of WTPT and PT6 by prothrombinase. Gels from SDS-PAGE time-course analysis of WTPT (●) and PT6 (○) activation by prothrombinase were quantified using densitometry to generate the time-course profiles of (A) prothrombin, (B) meizothrombin, (C) F1.2, and (D) B-chain of thrombin.

Table S1. Peptide Sequences.

*Cysteine in prothrombin sequence changed to serine in peptide.
¶F2P4 and F2P5 were insoluble.

Peptide Name	Prothrombin Residue Numbers	Peptide Sequence
F2P1	157-168	EGSSVNLSPPLE
F2P2	171-181	VPDRGQQYQGR
F2P3	179-190	QGRLAVTTHGLP
F2P4¶	191-205	LAWASAQAKALSKH
F2P5¶	206-217	QDFNSAVQLVEN
F2P6	232-242	YVAGKPGDFGY
F2P7	244-257	DLNYS*EEAVEEETG
F2P8	258-271	DGLDEDSDRAIEGR

References

1. Mann, K. G., Elion, J., Butkowski, R. J., Downing, M., and Nesheim, M. E. (1981) Prothrombin. *Methods Enzymol.* **80 Pt C**, 286–302
2. Nesheim, M. E., Prendergast, F. G., and Mann, K. G. (1979) Interactions of a fluorescent active-site-directed inhibitor of thrombin: dansylarginine N-(3-ethyl-1,5-pentanediyl)amide. *Biochemistry*. **18**, 996–1003
3. Bloom, J. W., Nesheim, M. E., and Mann, K. G. (1979) Phospholipid-binding properties of bovine factor V and factor Va. *Biochemistry*. **18**, 4419–25
4. Arni, R. K., Padmanabhan, K., Padmanabhan, K. P., Wu, T. P., and Tulinsky, A. (1994) Structure of the non-covalent complex of prothrombin kringle 2 with PPACK-thrombin. *Chem. Phys. Lipids*. **67–68**, 59–66
5. Church, W. R., Ouellette, L. A., and Messier, T. L. (1991) Modulation of human prothrombin activation on phospholipid vesicles and platelets using monoclonal antibodies to prothrombin fragment 2. *J. Biol. Chem.* **266**, 8384–8391
6. Luckow, E. A., Lyons, D. A., Ridgeway, T. M., Esmon, C. T., and Laue, T. M. (1989) Interaction of clotting factor V heavy chain with prothrombin and prethrombin 1 and role of activated protein C in regulating this interaction: analysis by analytical ultracentrifugation. *Biochemistry*. **28**, 2348–54

7. Xinyi Huang, X. (2003) Fluorescence Polarization Competition Assay: The Range of Resolvable Inhibitor Potency Is Limited by the Affinity of the Fluorescent Ligand. *J. Biomol. Screen.* **8**, 34–38
8. Kim, P. Y., and Nesheim, M. E. (2007) Further evidence for two functional forms of prothrombinase each specific for either of the two prothrombin activation cleavages. *J. Biol. Chem.* **282**, 32568–81
9. Koutychenko, A. (2002) *Interactions of human prothrombin fragment 2 with thrombin and factor Va*. Ph.D. thesis, McGill University
10. Li, X., Bokman, A. M., Llinás, M., Smith, R. A., and Dobson, C. M. (1994) Solution structure of the kringle domain from urokinase-type plasminogen activator. *J. Mol. Biol.* **235**, 1548–59
11. Byeon, I. J., Kelley, R. F., and Llinás, M. (1991) Kringle-2 domain of the tissue-type plasminogen activator. 1H-NMR assignments and secondary structure. *Eur. J. Biochem.* **197**, 155–65
12. Petros, A. M., Gyenes, M., Patthy, L., and Llinás, M. (1988) Analysis of the aromatic 1H NMR spectrum of chicken plasminogen kringle 4. *Arch. Biochem. Biophys.* **264**, 192–202
13. Arni, R. K., Padmanabhan, K., Padmanabhan, K. P., Wu, T. P., and Tulinsky, A. (1993) Structures of the noncovalent complexes of human and bovine prothrombin fragment 2 with human PPACK-thrombin. *Biochemistry*. **32**, 4727–4737
14. Papaconstantinou, M. E., Gandhi, P. S., Chen, Z., Bah, A., and Di Cera, E. (2008) Na+ binding to meizothrombin desF1. *Cell. Mol. Life Sci.* **65**, 3688–3697
15. Pozzi, N., Chen, Z., Pelc, L. A., Shropshire, D. B., and Di Cera, E. (2014) The linker connecting the two kringles plays a key role in prothrombin activation. *Proc. Natl. Acad. Sci. U. S. A.* **111**, 7630–7635
